# Phase I clinical trial of the bispecific antibody MDX-H210 (anti-Fc*γ*RI × anti-HER-2/neu) in combination with Filgrastim (G-CSF) for treatment of advanced breast cancer

**DOI:** 10.1038/sj.bjc.6601367

**Published:** 2003-12-09

**Authors:** R Repp, H H van Ojik, T Valerius, G Groenewegen, G Wieland, C Oetzel, B Stockmeyer, W Becker, M Eisenhut, H Steininger, Y M Deo, G H Blijham, J R Kalden, J G J van de Winkel, M Gramatzki

**Affiliations:** 1 Department of Medicine III, Division of Hematology/Oncology, University of Erlangen-Nürnberg, Krankenhausstraße 12, Erlangen 91054, Germany; 2 Department of Internal Medicine and Oncology. University Medical Center Utrecht, The Netherlands; 3 Immunotherapy Laboratory, Department of Immunology, University Medical Center Utrecht, The Netherlands; 4 Department of Gynecology, University of Erlangen-Nürnberg, Germany; 5 Department of Nuclear Medicine, University of Goettingen, Germany; 6 Department of Pathology, Hospital Friedrichshafen, Germany; 7 Medarex Inc., Annandale, New Jersey; 8 Genmab, Utrecht, The Netherlands; 9 Department of Nuclear Medicine, University of Heidelberg, Germany

**Keywords:** MDX-H210, Fc*γ*RI, HER-2/neu, G-CSF, immunotherapy

## Abstract

A phase I study of the bispecific antibody MDX-H210 in combination with granulocyte colony-stimulating factor (G-CSF) was performed in stage IV breast carcinoma patients, overexpressing HER-2/neu. MDX-H210, constructed by crosslinking antigen binding fragments (F(ab′) fragments) of monoclonal antibody (mAb) H22 to Fc gamma receptor I (Fc*γ*RI), and mAb 520C9 to HER-2/neu, respectively, mediates the lysis of tumour cells *in vitro*, and in human Fc*γ*RI transgenic mouse models. The proto-oncogene HER-2/neu is overexpressed in approximately 30% of breast cancer patients, and represents a promising target for antibody-based immunotherapy. Fc gamma receptor I (CD64) is an effective trigger molecule, which is expressed on monocytes/macrophages, immature dendritic cells, and G-CSF-primed polymorphonuclear cells (PMN). Patients received G-CSF (Filgrastim) for 8 consecutive days, and cohorts of three patients were treated on day 4 with escalating, single doses of MDX-H210. A total of 30 patients were included, and treatment was generally well tolerated, without reaching dose-limiting toxicity. Side effects consisted mainly of fever and short periods of chills, which were timely related to elevated plasma levels of interleukin 6 and tumour necrosis factor alpha. In the last two cohorts, MDX-H210 plasma levels exceeded 1 *μ*g ml^−1^, and on circulating myeloid cells >50% saturation of Fc*γ*RI was found until day 4. These effector cells were highly effective in antibody-dependent cell-mediated cytotoxicity. Immunohistochemical analyses of tumour biopsies in individual patients documented infiltration of monocytes and PMN after MDX-H210 infusion. Although the clinical course of the disease was not altered by the single dose of MDX-H210, a favourable toxicity profile – even at high doses – and remarkable biological effects were seen when combined with G-CSF. Therefore, the combination of G-CSF and MDX-H210 should be evaluated in further immunotherapeutical strategies.

Breast cancer represents a major public health problem in the Western world. In case of localised disease, cure rates have improved, but in women with metastatic disease results are still disappointing (Hortobagyi, 1998). Therefore, novel therapeutical approaches are under investigation. In the last decade, along with new chemotherapeutical drugs, developments in the field of immunology and genetic engineering have raised new perspectives. For example, Trastuzumab (Herceptin®), a humanised IgG1 monoclonal antibody (mAb) with specificity for HER-2/neu, has been approved for patients with metastatic breast cancer. As a single agent, the overall response rate was 15% in heavily pretreated patients with HER-2/neu overexpressing metastatic breast cancer (Cobleigh *et al*, 1999; Baselga, 2001).

The HER-2/neu gene encodes a 185–190 000 molecular weight transmembrane protein with tyrosine kinase activity. Overexpressed HER-2/neu has transforming activity, and in a variety of human carcinomas, gene amplification and protein overexpression have been demonstrated. This includes approximately 30% of breast cancer, in which it has been correlated with the development of metastases and is associated with poor prognosis (Slamon *et al*, 1987). In addition, HER-2/neu has limited expression on normal tissue, suggesting that it may constitute a valuable target for antibody therapy (Pegram and Slamon, 2000). Although many mechanisms have been proposed to account for the antitumour activities of therapeutical antibodies – including blockade of signalling pathways, activation of apoptosis, and antibody-dependent cell-mediated cytotoxicity (ADCC) – the relevance of these mechanisms in humans is still unclear (Houghton and Scheinberg, 2000). In mice, the significance of Fc receptor-bearing effector cells is clearly demonstrated by the reduced efficacy of Trastuzumab to arrest tumour growth in animals deficient in activating Fc receptors (Clynes *et al*, 2000).

Three different classes of leucocyte receptors for IgG (Fc*γ*R) have been distinguished in humans (van de Winkel and Capel, 1993; Ravetch and Bolland, 2001). Fc gamma receptor I (Fc*γ*RI) (CD64) is constitutively expressed on myeloid precursors in the bone marrow, monocytes/macrophages, immature dendritic cells, and can be induced on polymorphonuclear cell (PMN) by interferon gamma (IFN-*γ*) or granulocyte colony-stimulating factor (G-CSF) (Perussia *et al*, 1983; Repp *et al*, 1991). Fc*γ*RI is unique among Fc*γ*R because of its high affinity for IgG, the limited cell expression solely on cytotoxic effector cells, and because of its inherent capacity to trigger cytotoxic effector cells. Furthermore, Fc*γ*RI contains a distinct cytoplasmatic domain, which targets antigens to MHC class II-containing vesicles, and thereby, enhances antigen presentation (van Vugt *et al*, 1999).

Bispecific antibodies (BsAbs) can effectively link target cells to cytotoxic trigger molecules on immune effector cells, and may overcome some of the limitations of conventional mAb (Segal *et al*, 1999). For example, high concentrations of serum immunoglobulins compete *in vivo* for binding to Fc*γ*RI on immune effector cells. Hereby, the ability of therapeutical antibodies to trigger Fc*γ*RI could be impaired. To overcome this problem, the BsAb MDX-H210 was constructed by chemical crosslinking of F(ab′) fragments from humanised mAb 22 (H22), directed to Fc*γ*RI, and mAb 520C9, to the proto-oncogene product HER-2/neu, respectively. Monoclonal antibody H22 binds to a site distinct from the ligand-binding site, yet effectively triggers Fc*γ*RI-mediated cellular responses (such as phagocytosis, respiratory burst, and ADCC) in the presence of high concentrations of human serum IgG (Guyre *et al*, 1989; Valerius *et al*, 1993). To influence the effector cell system specifically, G-CSF was added to the treatment schedule to increase the absolute numbers of PMN, to induce the expression of Fc*γ*RI (Repp *et al*, 1991), and to enhance their functional capacity (Valerius *et al*, 1993). Preclinical studies demonstrated the binding of MDX-H210 to Fc*γ*RI on G-CSF-primed PMN and to HER-2/neu on SK-BR-3 breast carcinoma cells to be comparable to the respective parental mAb. More importantly, *in vitro* MDX-H210 was highly cytotoxic to HER-2/neu overexpressing cell lines, when Fc*γ*RI-positive PMN from G-CSF-treated patients served as effector cells (Stockmeyer *et al*, 1997; Stockmeyer *et al*, 2001). In addition, studies in an Fc*γ*RI-transgenic animal model reported synergism of Fc*γ*RI-directed BsAb and G-CSF, resulting in antitumour activity and in ‘memory’ induction (Honeychurch *et al*, 2000).

To analyse whether these preclinical observations may translate into the clinical situation, MDX-H210 in combination with G-CSF was tested in a phase I study. The primary objectives of this trial were to define the maximum tolerated dose (MTD), and to identify the optimal biologically active dose of MDX-H210. The secondary objective was to assess the clinical responses.

## PATIENTS AND METHODS

### Patients

Patients, aged 18–70 years, with stage IV breast cancer were eligible if their primary tumour or metastases were overexpressing HER-2/neu. The expression of HER-2/neu was determined on paraffin-embedded tissues using standard immunohistochemical methods (Venter *et al*, 1987). Inclusion in the trial demanded a 2+ or 3+ membrane staining (van de Vijver, 2001). Patients needed to be progressive after at least two standard chemotherapy or hormonal regimens, or one hormonal and one chemotherapy regimen. Sufficient blood cell counts, normal hepatic, and renal function, as well as an Eastern Cooperative Oncology Group performance status of <3, and a life expectancy of more than 3 months were required. The protocol was approved by the local Ethics Committee, and written informed consent before treatment was obligatory.

### Study design

Bispecific antibody MDX-H210 was given as a single, 2-h intravenous (i.v.) infusion, in combination with a fixed subcutaneous (s.c.) dose of 5 *μ*g kg^−1^ Filgrastim for 8 days in the afternoon, with a minimum of 4 h after the end of MDX-H210 on day 0 (Table 1

**Table 1:** Treatment schedule

**Day**	**−3**	**−2**	**−1**	**0**	**1**	**2**	**3**	**4**
Filgrastim s.c. (5 *μ*g kg^−1^)	x	x	x	x	x	x	x	x
MDX-H210 i.v. (dose escalation^a^)				x				

a MDX-H210 is given as a single 2 h infusion with escalating doses from 0.35 up to 200 mg m^−2^.

). Filgrastim was started 3 days before the administration of MDX-H210 to ensure maximal expression of Fc*γ*RI on neutrophils (Repp *et al*, 1991). The dose of MDX-H210 was escalated in cohorts of three patients, and not in an individual patient. For the first cohort of patients, Filgrastim was skipped if the absolute neutrophil count (ANC) was higher than 20 000 *μ*l^−1^ to prevent toxicity. As no toxicity was observed, the upper limit for ANC was raised to 50 000 *μ*l^−1^ from the second cohort onwards. In the first two cohorts, the same dose of MDX-H210 (0.35 mg m^−2^) was given. Patients treated in the third to tenth cohort received escalating doses of MDX-H210 from 1 up to 200 mg m^−2^ (1, 3.5, 7, 10, 15, 30, 100, and 200 mg m^−2^). Dose-limiting toxicity (DLT) was defined as the dose where three or more patients in a cohort of six patients experienced grade III or IV toxicity, and the MTD as the dose level below which DLT occurred. Reassessment for tumour response was performed after 30 days. If patients had an objective response or stable disease, a second treatment on compassionate use basis was possible for patients enrolled during the last period according to an amendment. The overall goals of this phase I trial were to determine toxicity, MTD, pharmacokinetics, and pharmacodynamics of MDX-H210, to describe the biological effects of MDX-210 in combination with Filgrastim, and to elucidate the effects of Filgrastim on immune effector cells.

### Production of MDX-H210

Bispecific antibody MDX-H210 (Fc*γ*RI × HER-2/neu) was produced by chemically crosslinking F(ab′) fragments of mAb 22 against Fc*γ*RI (CD64) with mAb 520C9 against HER-2/neu, as described by Glennie *et al* (1987). Briefly, F(ab′*γ*)_2_ fragments were produced by limited proteolysis with pepsin, and were then reduced to provide F(ab′*γ*) with free hinge region SH groups. The SH groups on one of the F(ab′*γ*)-SH partners were then fully alkylated with excess *o*-phenylendimaleimide (*o*-PDM) to provide free maleimide groups. Finally, the two *o*-PDM and F(ab′)-SH preparations F(ab′) were combined at a ratio of 1 : 1 to generate heterochimeric constructs. After purification by size exclusion chromatography, the binding activity of the individual components was verified by flow cytometry, using HER-2/neu-expressing SK-BR-3 cells, Fc*γ*RI-expressing human monocytes, and U937 cells as targets. The bispecific nature of the construct was confirmed by a solid-phase immunoassay. MDX-H210 was produced according to GMP guidelines by Medarex (Medarex Inc., Annandale, NJ, USA), and shipped as a clear colourless liquid in sterile phosphate-buffered saline for i.v. infusion.

### Assessment of adverse reactions

Patients were hospitalised on day −1 and MDX-H210 was administered as a 2-h i.v. infusion on day 0 (Table 1). The vital signs were frequently monitored during and for 24 h after the administration of the BsAb. Afterwards, patients were checked on an outpatient basis. Laboratory tests were performed daily starting on day −3 with additional time points 2, 4, and 8 h after the start of MDX-H210 infusion. Toxicity was graded according to the NCI Common Toxicity Criteria. For dose escalation, transient leucopenia, neutropenia without signs of infection, or fever with mild hypotension were not graded as toxicity, because these symptoms were known to be correlated to MDX-H210 infusion, and to recover spontaneously (Valone *et al*, 1995).

### Pharmacokinetics

Blood samples were obtained before and 2, 4, 8, 24, 48, 72, and 96 h after BsAb infusion. Plasma was immediately separated and stored at −70°C until analysis. Microtitre plates, coated with goat antimurine IgG, were incubated with serial dilutions of the patient's plasma, or with MDX-H210 diluted in normal human plasma (Nabi Inc., Boca Raton, FL, USA). The captured BsAb was detected by an alkaline phosphatase-conjugated antimurine IgG. Pharmacokinetic analysis was performed using PKPD Tools for Excel Version 1.02 (Charles Minto, Stanford, CA, USA) with a constant i.v. infusion open noncompartmental model.

### Cytokines, tumour markers, and plasma HER-2/neu levels

Plasma – separated and stored as described above – was analysed for levels of interleukin (IL)-1*α*, IL-1*β*, IL-2, IL-6, IL-10, IFN-*γ*, tumour necrosis factor alpha (TNF-*α*), G-CSF, soluble IL-2 receptor (sIL-2R), CEA, CA125, and CA15.3 by enzyme-linked immunosorbent assay using kits according to the manufacturer's instructions (R&D Systems, Minneapolis, MN, USA). Plasma HER-2/neu was measured by enzyme immunosorbent assay using a commercial kit (Chiron Diagnostics, Alameda, CA, USA).

### Radioimaging with Technetium-99m (^99m^Tc) MDX-H210

Antibody labelling was performed according to a published kit-formulated NHS-BAT (NHS=*N*-hydroxysuccinimide and BAT=*bis*(aminoethanethid)type) ester conjugation (Eisenhut *et al*, 1996). Briefly, NHS ester of the BAT ligand, 6-(4′(4″-carboxyphenoxy)butyl)-2,10-dimercapto-2.10-dimethyl-4,8-dizaun-decane was conjugated with MDX-H210 at ambient temperature, and pH ranging from pH 7.0 to pH 8.5. Unreacted or hydrolysed NHS-BAT ester was separated by one or two ultracentrifugation steps (for 15 min each). The conjugation yield was 70–80% independent of pH. Complexation of the BsAb conjugate with ^99m^Tc was accomplished using Sn(II)-tartrate as a reducing agent. Within 5 min, labelling yield >95% was achieved using 1 mg BAT-MDX-H210 comprising about three BAT ligands per antibody, and up to 1.5 GBq ^99m^Tc-pertechnetate. Localisation of MDX-H210 in normal and tumour tissues was investigated in a subset of patients who voluntarily participated, using planar whole-body scans, and single photon emission tomography (SPECT) images of the regions of interest.

### Granulocyte scintigraphy with Indium-111-oxine (^111^In)-labelled PMN

Autologous granulocytes were isolated using a Percoll/plasma gradient, and labelled with ^111^In-oxine as described (Becker *et al*, 1986). Labelled granulocytes were reinfused either 30 min before, or 72 h after application of MDX-H210. Dynamic imaging was performed with a gamma camera linked to a computer system during 2 h after injection of labelled cells to trace the route of PMN.

### Human antibispecific antibody (HABA) response

Microtitre plates coated with MDX-H210 were incubated with dilutions of plasma samples. Anti-MDX-H210 antibodies were detected with an alkaline-phosphatase-conjugated goat anti-human IgG Fc-specific probe. The dilution of plasma required to reach the background level (*A*_405_=0.1) was determined for samples before infusion, and on days 7, 14, and 30 postinfusion. Human antibispecific antibody levels were expressed as *x*-fold increase over the baseline preinfusion value.

### Assessment of biological effects

#### Isolation of effector cells

A measure of 10–20 ml of peripheral blood was drawn from patients or healthy donors (HDs) after informed consent. The total leucocyte counts in whole blood were determined on an EPICS™ Profile flow cytometer (Coulter, Hialeah, FL, USA), and PMN counts in whole blood were calculated from the total leucocyte counts and differentials of Wright-stained blood smears. Isolated PMN were obtained by a method slightly modified from that described in Repp *et al* (1991). Briefly, citrate anticoagulated blood was layered over a discontinuous Percoll (Seromed, Berlin, Germany) gradient consisting of 70 and 62% for HD, and of 68 and 59% of Percoll for Filgrastim recipients, respectively. After centrifugation, PMN were collected at the interphase between the two Percoll layers, and mononuclear cells from the Percoll/plasma interphase. The remaining erythrocytes were removed by hypotonic lysis. The purity of PMN was determined by cytospin preparations and exceeded 95%, with few contaminating eosinophils and <1% mononuclear cells. The viability of cells tested by Trypan blue exclusion was always exceeding 95%.

#### Immunofluorescence

Antibodies 32.2 (Fc*γ*RI, CD64; mIgG1) (Guyre *et al*, 1989), IV.3 (Fc*γ*RII, CD32; mIgG2b) (Looney *et al*, 1986), 3G8 (Fc*γ*RIII, CD16; mIgG1) (Fleit *et al*, 1982), and 520C9 (HER-2/neu; mIgG1) (Ring *et al*, 1989) were obtained from Medarex Inc. (Annandale, NJ, USA). Whole blood (100 *μ*l) was incubated with different lineage-specific FITC- or RPE-conjugated antibodies (CD3, CD4, CD8, CD20, CD11b, CD56, CD67) for 30 min at 4°C. Afterwards, samples were subjected to FACS® Lysing solution to lyse red blood cells and fix white cells. Cells were then washed three times in phosphate-buffered saline supplemented with 1% bovine serum albumin, and analysed on an EPICS™ Profile flow cytometer (Coulter). For each cell population, the relative fluorescence intensity (RFI) was calculated as the ratio of mean linear fluorescence intensity of relevant to irrelevant, isotype-controlled antibodies.

#### ADCC assay

Antibody-dependent cell-mediated cytotoxicity was performed as described (Valerius *et al*, 1993). Briefly, human SK-BR-3 breast carcinoma cells were used as the target. This cell line was obtained from the American Type Culture Collection (Manassas, VA, USA), and kept in an RF10^+^ medium consisting of RPMI 1640 medium (Gibco BRL, Paisley, Scotland) supplemented with 10% heat-inactivated foetal calf serum, 100 U ml^−1^ penicillin, 100 U ml^−1^ streptomycin, and 4 mM L-glutamine (all Gibco BRL). The target cells were labelled with 200 *μ*Ci ^51^Cr for 2 h. After extensive washing with RF10^+^, cells were adjusted to 10^5^ ml^−1^. Isolated PMN, effector-to-target ratio 80 : 1 (E : T, 80 : 1), or 50 *μ*l of whole blood as effector source, sensitising antibodies and RF10^+^ were added into round-bottom microtitre plates. By adding the target cell suspension, giving a final volume of 200 *μ*l, the assays were started. After 3 h at 37°C, assays were stopped by centrifugation, and ^51^Cr release from triplicates was measured in counts per minute (c.p.m.). The percentage of cellular cytotoxity was calculated using the formula:


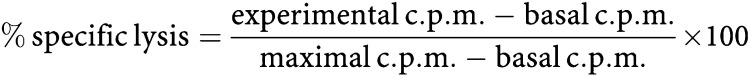


with maximal ^51^Cr release determined by adding perchloric acid (3% final concentration) to target cells, and basal release measured in the absence of sensitising antibodies and effector cells. Only very low levels of antibody-mediated noncellular cytotoxicity (without effector cells) were observed under these assay conditions (<5% specific lysis). Low levels of antibody-independent killing were seen in whole-blood assays (Figure 6).

#### Oxidative burst and phagocytosis

The activity of neutrophil respiratory burst was measured by a method modified from Smith and Weidemann (1993). In short, 100 *μ*l of whole blood was incubated for 15 min with 1 *μ*l of 2′,7′-dichlorofluorescein diacetate (200 mM solution in ethanol; Molecular Probes, Eugene, OR, USA). After stimulation for 15 min with 1 *μ*g ml^−1^ PMA (Sigma, Deisenhofen, Germany), red cell lysis and fixation of white cells was obtained by Q-prep (Coulter). Fluorescence of single cells was measured on an EPICS™ Profile flow cytometer (Coulter), and the results were analysed comparing mean fluorescence intensity.

For measurement of phagocytosis, fluorescent polystyrene beads (1.0 *μ*m in diameter; Fluoresbrite, Polysciences Inc., Warrington, PA, USA) were opsonised with human albumin or polyclonal human IgG, respectively, according to the manufacturer's instructions for coupling proteins to carboxylated polystyrene microparticles (Carbodiimide method, Polysciences Inc., Warrington, PA, USA). A measure of 2 *μ*l of 2.5% carboxylated microparticles were added to 100 *μ*l whole blood, and incubated for 30 min at 37°C. Red cell lysis and fixation of white cells was obtained by Q-prep, and the fluorescence of single cells was measured on an EPICS™ Profile flow cytometer (Coulter). Polymorphonuclear cell populations were gated according to forward light scatter and perpendicular light scatter. Data were expressed as the percentage of PMN-containing FITC-labelled beads and the mean number of beads per PMN (calculated from the fluorescence intensity of PMN with a single particle ingested).

### Statistical analysis

Group data are reported as mean±standard deviation (s.d.). The differences between groups were analysed by unpaired (or, when appropriate, paired) Student's *t*-tests. Levels of significance are indicated. Data presented correspond to the whole patient population, or is indicated when different.

## RESULTS

### Patient characteristics

In all, 30 women with metastatic breast cancer were treated in this trial. Three patients were treated in each of 10 cohorts received escalating doses of MDX-H210 from 0.35 up to 200 mg m^−2^. The first and second cohorts were treated with 0.35 mg m^−2^ with an upper limit of the ANC of 20 000 *μ*l^−1^ in the first and 50 000 *μ*l^−1^ from the second cohort onwards. Their ages ranged from 40 to 69 years, with a median age of 50 years. The median time from diagnosis was 70.2 months. Patients were heavily pretreated, but had not received previous antibody therapy (Table 2

**Table 2:** Patient characteristics

*Patient demographics*	
Total number	30
Age (years)	50 (40–69)
Median time since diagnosis (months)	70.2 (7.1–181.6)
Metastatic sites	2 (1–5)
	
*Prior therapy*	
Chemotherapy	2 (0–6)
Radiotherapy	1 (0–7)
Hormonal treatment	2 (0–4)
	
*ECOG*	
0	4
1	20
2	6

).

### Pharmacokinetics

**Figure 1:**
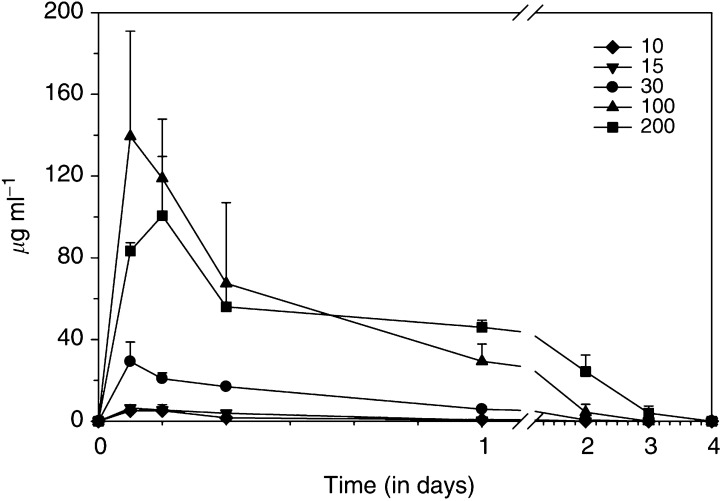
Plasma levels of MDX-H210. Plasma levels of MDX-H210 in the different patient cohorts were determined 2, 4, 8, 24, 48, 72, and 96 h after BsAb infusion. The related pharmacokinetic parameters are described in Table 3. Data presented relate to all participating patients treated with ⩾10 mg m^−2^.

Plasma levels of MDX-H210 were measured up to 4 days after BsAb infusion (Figure 1

). The mean *t*_1/2_ values ranged from 4.76 h in the 3.5 mg m^−2^ cohort to 17.41 h in the 200 mg m^−2^. *C*_max_ values were correlated with the applied dose, and varied from 0.24 mg l^−1^ at the 1 mg m^−2^ level to 155.7 mg l^−1^ at the 100 mg m^−2^ dose level (Table 3

**Table 3:** Plasma pharmacokinetics

**Dose level^a^(mg m^−2^)**	***t*_1/2_ (h)**	***C*_max_^b^(*μ*g ml^−1^)**	***T*_max_ (h)**	**AUC^c^(*μ*g h^−1^ ml^−1^)**	***β*^d^**	**Vd^e^(l kg^−1^)**	**Cl^f^(ml min^−1^ kg^−1^)**
1	—	0.24	2	—	—	—	—
3.5	4.76	1.28	2.7	9.33	0.180	0.070	0.747
7	5.19	5.95	2	41.90	0.137	0.036	0.300
10	10.54	5.82	3.3	65.22	0.078	0.063	0.269
15	6.09	6.72	2.7	70.72	0.117	0.052	0.417
30	9.00	29.34	2.7	381.06	0.078	0.027	0.134
100	9.53	155.27	2.7	1937.77	0.073	0.020	0.091
200	17.41	109.00	4	2523.08	0.041	0.049	0.147

a Pharmacokinetic results could not be determined accurately for dose level 0.35 mg m^−2^ because low plasma concentrations of MDX-H210 resulted in very few measurements.

b Maximal concentration.

c Area under the curve.

d Elimination rate constant *β*.

e Volume of distribution.

f Plasma clearance.

). At the 200 mg m^−2^ dose level, infusion of MDX-H210 was temporarily stopped in two patients because of chills and vomiting during the infusion period and restarted after about 1 h. Owing to the prolonged infusion time, *C*_max_ was lower compared to the 100 mg m^−2^ dose level and was reached after 4 h (Table 3). Both granulocytes and monocytes were opsonised with MDX-H210 at the end of the infusion, and continued to be ‘armed’ with MDX-H210 up to 4 days after the infusion of 200 mg m^−2^ (Figure 2

**Figure 2:**
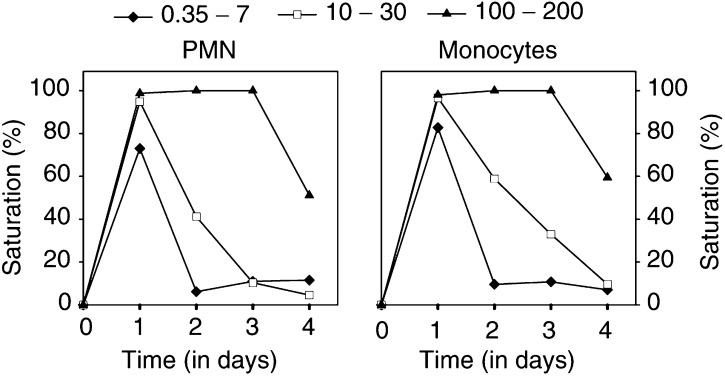
Cell-bound MDX-H210. The total number of Fc*γ*RI expressed by circulating cells was determined after incubation with a saturating dose of MDX-H210. Patients were divided into three groups according to treatment with low (0.35–7 mg m^−2^), medium (10–30 mg m^−2^), and high (100–200 mg m^−2^) doses of MDX-H210. Cell-bound murine IgG without or with this preincubation was determined by incubation with FITC-labelled goat anti-mouse mAb. Significant amount of bound MDX-H210 was found on monocytes and PMN on day 1 in the low-dose group and days 1–4 in the medium- and high-dose group (*P*<0.05).

), with a comparable expression of Fc*γ*RI (CD64) on days 0 and 3 (Figure 4).

### Toxicity

In the current study, the combination of MDX-H210 with Filgrastim was well tolerated with minimal to moderate toxicity up to doses of 200 mg m^−2^ (Table 4

**Table 4:** Maximum nonhaematological toxicity

	**Cohort (mg m^−2^)/toxicity (grade)**																	
**Dose level (number of patients)**	**0.35 (*n*=6)**		**1 (*n*=3)**		**3.5 (*n*=3)**		**7 (*n*=3)**		**10 (*n*=3)**		**15 (*n*=3)**		**30 (*n*=3)**		**100 (*n*=3)**		**200 (*n*=3)**	
**Toxicity grade**	**1/2**	**3**	**1/2**	**3**	**1/2**	**3**	**1/2**	**3**	**1/2**	**3**	**1/2**	**3**	**1/2**	**3**	**1/2**	**3**	**1/2**	**3**
Nausea	3		2		2		3		3		2		3		2		1	1
Vomiting	1		2		2		3		3		1		3		3		1	1
Fever	3		3		3		3		3		3		3		3		2	
Chills	3		3		2		1		1		1		2		1		1	
Hepatic	5		3		3	1	3		3		2		1		2		1	
Respiratory	1				1		1		1			1			1	1	1	
Tachycardia	1				1		2				1				2		2	
Hypotension	1				1				1				1		3		2	
Tumour pain	2		2		1				2		1		1		3		1	
Redness of skin	1		1								1							
Diarrhoea													1		1			
Emotional			1		1													

). No grade IV toxicity occurred. Nonhaematological toxicities consisted mainly of short periods of chills, low-grade fever, nausea, and vomiting all occurring towards the end of the MDX-H210 infusion. Most side effects resolved spontaneously within 8 h, or could easily be treated with either acetaminophen or antiemetics. Several patients complained about pain at metastatic sides, possibly related to local inflammatory reactions in tumour tissue induced by the BsAb, or to therapy-related cytokine release. A one- to two-fold increase of alkaline phosphatase and *γ*GT was noted, which was maximal after 3–4 days, and resolved spontaneously after 1 week. Haematological changes were mainly related to the treatment medication. After the start of Filgrastim an asymptomatic granulocytosis emerged. At 2 h after infusion of MDX-H210, granulocytes and monocytes decreased dramatically. However, these values returned to preinfusion levels within approximately 12 h. Lymphocytes, which do not express Fc*γ*RI, also demonstrated a significant drop after infusion of MDX-H210. Red blood cell counts and platelet numbers did not significantly change (Figure 3

**Figure 3:**
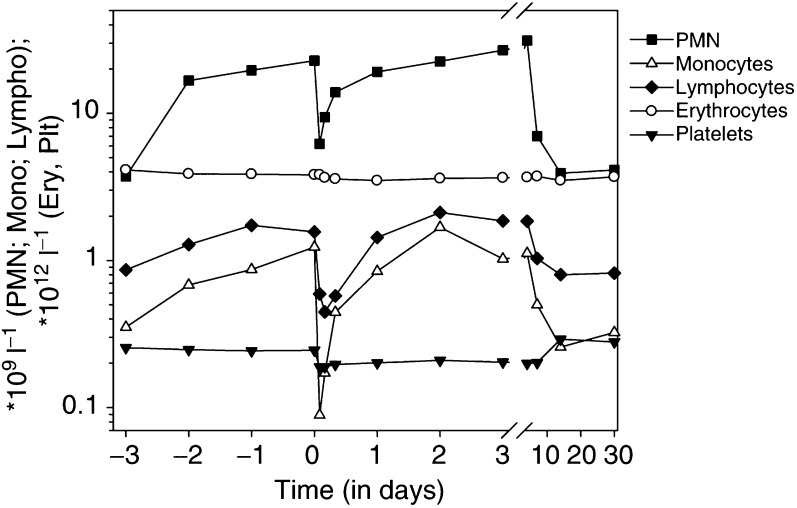
Effects of MDX-H210 on circulating white blood cells numbers. In all patients, ANC increased during Filgrastim application. A significant drop of ANC, monocytes, and lymphocytes was observed after infusion of MDX-H210 with a minimum 2–4 h after start of BsAb infusion. Data presented relate to all participating patients.

).

### Receptor expression

**Figure 4:**
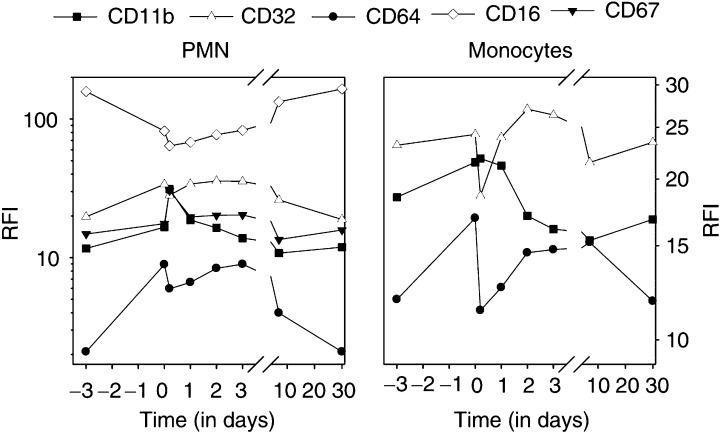
Receptor expression of PMN and monocytes during treatment with MDX-H210/Filgrastim. Receptor expression was determined by flow cytometry and expressed as RFI. Changes from days −3 to 0 are attributed to the effect of Filgrastim; further changes were noted after start of the 2 h during infusion of MDX-H210 on day 0. Data presented relate to all participating patients. ^**^Significant changes (*P*<0.05) were seen for all tested antigens between days −3 and 0 and for all, except CD11b on monocytes between day 0 and the end of MDX-H210 infusion. No significant differences were seen between days −3 and 30.

At 72 h after the start of Filgrastim, all blood PMN expressed Fc*γ*RI (CD64), declining slowly after G-CSF cessation (Figure 4

). A small increase of Fc*γ*RI expression was seen on monocytes. Within 2 h after the start of MDX-H210, a transient decrease of Fc*γ*RI expression was seen on monocytes and PMN, suggesting a more rapid decline of Fc*γ*RI-positive cells. Interestingly, Fc*γ*RII (CD32) expression on monocytes and PMN was also increased during Filgrastim application, whereas Fc*γ*RIII (CD16) expression of PMN significantly decreased. The expression of CD67 on PMN, and of CD11b on PMN and monocytes increased after the administration of MDX-H210, probably due to concomitant cytokine release.

### Functional assays

#### ADCC, phagocytosis, and oxidative burst

**Figure 5:**
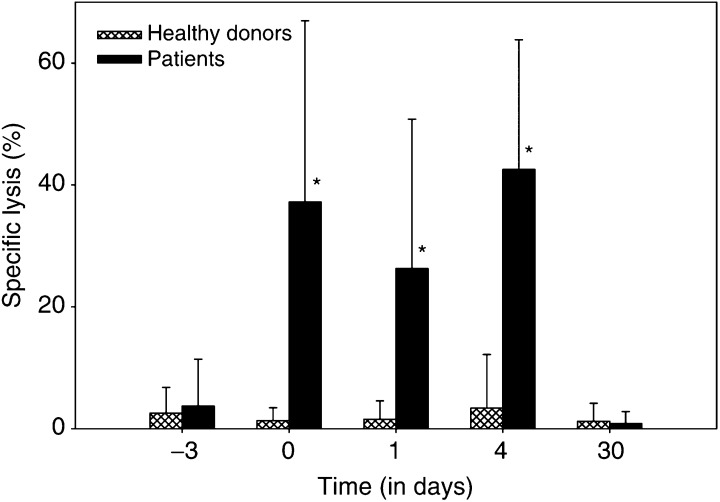
ADCC of isolated PMN via MDX-H210. Isolated PMN from HD were compared with PMN from patients in their capacity to mediate cytotoxicity against SK-BR-3 breast cancer cells in the presence of 0.4 *μ*g ml^−1^ MDX-H210 at an E : T ratio of 80 : 1. Lysis of SK-BR-3 by PMN from patients on days 0, 1, and 4 is significantly higher compared to day −3 (before start of Filgrastim) as well as compared to healthy controls (*P*<0.005, indicated by ^*^). Data presented (mean±s.d.) relate to a subset of 26 patients and 26 HDs.

*In vitro* cytotoxicity assays against SK-BR-3 breast cancer cells with isolated PMN demonstrated significantly enhanced cytotoxicity in the presence of MDX-H210 during, but not before or 1 week after the start of Filgrastim application (Figure 5

). A small decrease in ADCC activity of PMN on day 1 probably reflected the reduced Fc*γ*RI expression on the remaining circulating PMN.

**Figure 6:**
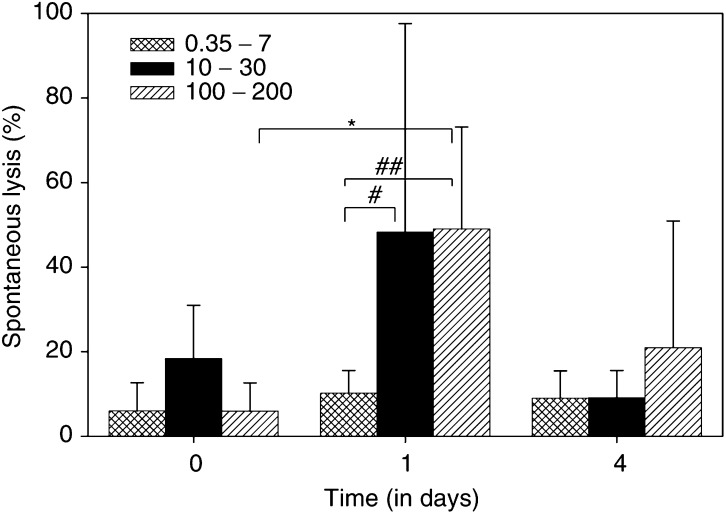
Spontaneous cytotoxicity. For whole-blood ADCC, citrate anticoagulated, freshly drawn blood was added to ^51^Cr-labelled SK-BR-3 cells without the addition of antibody. Patients were divided into three groups according to treatment with low (0.35–7 mg m^−2^), medium (10–30 mg m^−2^), and high (100–200 mg m^−2^) doses of MDX-H210. Significant enhanced lysis was seen in patients of the high-dose group (day 1 *vs* day 0, *P*<0.05, indicated by ^*^). The medium- and high-dose group showed enhanced lysis compared to the low-dose group on day 1 (#: *P*<0.05; ##: *P*<0.01). Patients treated with high doses of MDX-H210 (⩾100 mg m^−2^) showed a trend toward enhanced lysis of SK-BR-3 even 4 days after MDX-H210 infusion, although not statistically significant.

Spontaneous cytotoxicity of whole blood, without the addition of MDX-H210 *in vitro* could be demonstrated on day 1 in cohorts treated with doses above 10 mg m^−2^, lasting up to day 4 with doses above 100 mg m^−2^ (Figure 6

). This spontaneous cytotoxicity documented adequate circulating MDX-H210 levels to induce ADCC, and was in agreement with the measurement of cell-bound MDX-H210 (Figure 2).

**Figure 7:**
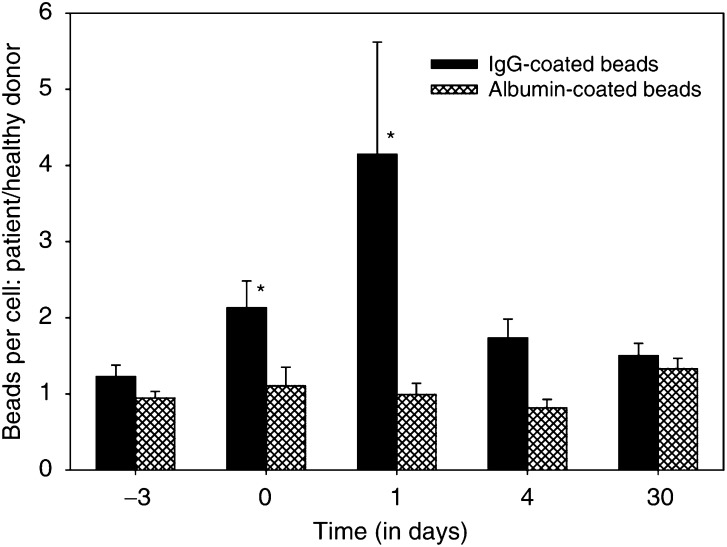
Phagocytosis of latex beads by PMN during treatment with MDX-H210. Phagocytosis of 1.0 *μ*M fluorescent polystyrene beads, coated with human albumin and polyvalent human IgG, respectively, was measured by flow cytometry. Mean beads per neutrophil from a patient and an HD measured simultaneously were calculated as described in Material and Methods. The relation of beads per PMN of patient to HD is expressed. Phagocytosis of IgG-coated beads is significantly higher on days 0 and 1 (*P*<0.05, indicated by ^*^) compared to baseline (day −3).

Phagocytosis of IgG-coated beads by PMN was increased during Filgrastim application, with a further increase 24 h after MDX-H210 infusion. In contrast, phagocytosis of albumin-coated beads did not change (Figure 7

).

Baseline oxidative burst, unstimulated and stimulated with PMA, was comparable to HDs (60.5±22.1 *vs* 59.8±29.0 and 151.5±25.8 *vs* 149.6±25.7, respectively). A slight increase of PMA-stimulated oxidative burst was seen during Filgrastim (158.3±23.2; *P*=0.07), which increased to significant values 24 h after the application of MDX-H210 (163.7±21.6; *P*=0.01), returning to baseline on day 4.

#### Cytokines, HABA

**Figure 8:**
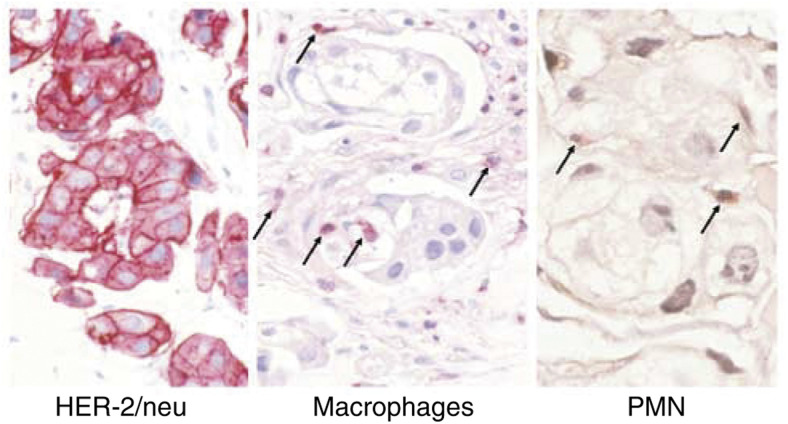
Cytokine plasma levels. Mean plasma levels of all patients are shown. Plasma levels of IL-6 and TNF-*α* increase after the infusion of MDX-H210 with a maximum 2 h after the start of infusion. Anti-inflammatory cytokine IL-10 is released with a similar time kinetic. G-CSF levels begin to rise after the start of Filgrastim therapy, with a further significant increase after MDX-H210. Plasma levels of sIL-2R and G-CSF were significantly different between days −3 and 0 (*P*<0.001). Significant differences (*P*<0.05) of plasma levels before (day 0) and after infusion of MDX-H2120 were seen for TNF-*α* (8 h and 1 day after start of MDX-H210), IL-6 (2 h and 1 day after start of MDX-H210), and sIL-2R (from 2 h up to 3 days after start of MDX-H210). No significant release of IL-1*α*, IL-1*β*, IL-2, IFN-*γ*, and IL-12 was seen.

Cytokine plasma concentrations were measured at baseline, and at several time points during the study. Elevated levels of IL-6 and TNF-*α* were consistently found during the first hour after MDX-H210 infusion (Figure 8

), and related to flu-like symptoms. Peak levels of TNF-*α* and IL-6 did not correlate to the dose of BsAb applied. Whereas peak levels of TNF-*α* were reached after 2 h, IL-6 levels were maximal after 4 h. The anti-inflammatory cytokine IL-10 also increased, with a maximum after 2 h. Granulocyte colony-stimulating factor plasma levels increased during the application of Filgrastim, with a small, additional increase after MDX-H210 infusion. Soluble IL-2 receptor increased after the start of Filgrastim, and reached its maximum after MDX-H210. Serum levels of IFN-*γ*, IL-1*α*, IL-1*β*, IL-2, and IL-12 were not altered during the study period. Human antibispecific antibody was detected in 19 patients on day 30, in seven of them ⩾16 times above baseline.

#### Scintigraphy

The number of circulating PMN rapidly decreased after the tart of MDX-H210 infusion. In order to evaluate whether G-CSF-activated PMN could cause additional toxicity, for example, by trapping of PMN in the lung, dynamic granulocyte imaging was performed with ^111^In-labelled autologous granulocytes infused 30 min prior to MDX-210 infusion. A normal distribution of granulocytes with no abnormal accumulation at any particular site was found (patient #3). In two patients, either ^111^In-labelled (patient #12) or ^99m^Tc-HMPAO-labelled autologous granulocytes (patient #23) were infused 72 h after the administration of MDX-H210. Imaging of the liver, spleen, and bone marrow was normal. However, sites of bone metastasis were spared (patient #12), and soft-tissue metastases did not image well. In three patients (patient #6, #11, and #28), 200 *μ*g of ^99m^Tc-labelled MDX-H210 was injected simultaneously with unlabelled antibody, resulting in imaging of the liver, spleen, and bone marrow, but without imaging tumour sites. In patient #28, however, asymptomatic brain metastases were detected by SPECT. Since these lesions were also seen with ^99m^Tc-DTPA SPECT without antibody, a nonspecific imaging due to an altered blood–brain barrier has to be considered. In all three patients, activity was detected in the gut, suggesting excretion of labelled MDX-H210 via the bile duct.

### Response

**Figure 9:**
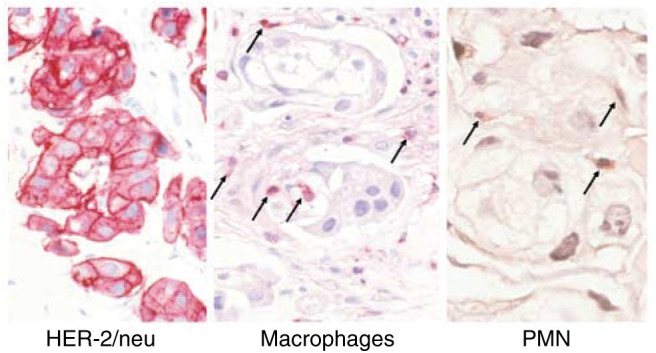
Immunohistochemical staining of a skin biopsy. A skin biopsy taken 3 days after the administration of MDX-H210 (patient #21) was stained for macrophages and PMN. Macrophages (arrow) were stained with a CD68 antibody, and PMN (arrow) with chloroacetate esterase. A skin biopsy from the same patient taken before the start of treatment showed no relevant infiltration with macrophages and PMN.

**Figure 10:**
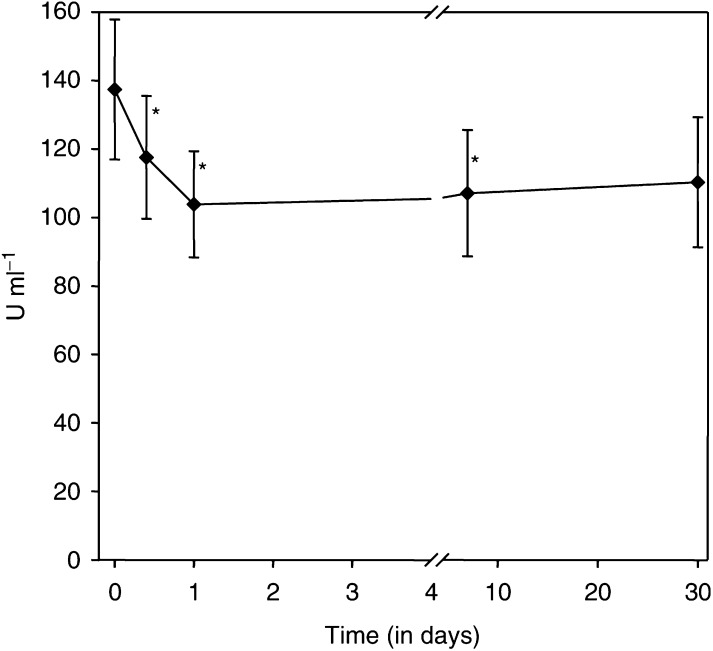
Plasma levels of soluble HER-2/neu. Soluble HER-2/neu levels were measured during treatment with MDX-H210 and Filgrastim. A significant decrease of sHer-2/neu was seen between 4 h after the start of infusion and 7 days (*P*<0.05). Data are presented as mean±s.e.m. of all patients.

No objective responses were seen in this single dose study in advanced patients. A total of 11 patients had stable, and 19 patients progressive disease at the end of the study period. Three patients with metastatic skin lesions developed an erythema between days 1 and 4 after the administration of MDX-H210. In one of them, a skin biopsy was performed before and 3 days after MDX-H210 application. Immunohistochemical analyses revealed local infiltration of mononuclear cells and PMN at the tumour site (Figure 9

). A control biopsy before the study showed no effector cell infiltration. Patient #29 (200 mg m^−2^) demonstrated a reduction of CA 15.3–40.2% (341 *vs* 137 E ml^−1^) and CEA to 37.8% (64.9 *vs* 24.1 UG l^−1^) on day 30, increasing later again. Soluble HER-2/neu levels increased 4 h after infusion of MDX-H210 in average by 25.2±23.2 U ml^−1^ (*P*=0.045) in patients treated with 0.35 mg m^−2^, whereas an early drop was seen in most of the patients treated with higher doses (mean decrease of 32.9±36.7 U ml^−1^; *P*<0.001). The mean decrease of all patients was maximal on day 7 (mean decrease of 34.8±79.0 U ml^−1^; *P*=0.034), while on day 30 this was less impressive (a mean decrease of 26.2±76.8 U ml^−1^; *P*=0.083) (Figure 10

).

## DISCUSSION

Based on our preclinical data (Stockmeyer *et al*, 1997), the combination of MDX-H210 and G-CSF was analysed in a phase I study, performed in 30 heavily pretreated breast carcinoma patients overexpressing HER-2/neu. This phase I trial was designed to determine the tolerability, safety, and MTD of BsAb MDX-H210 (anti-Fc*γ*RI × anti-HER-2/neu) in combination with Filgrastim. Aside from clinical toxicity, we focused on the biological activity, and pharmacokinetics of the combination. Treatment was generally well tolerated, with a successful dose escalation of MDX-H210 up to 200 mg m^−2^ without reaching MTD. Nonhaematologic toxicity appeared within the first 2–4 h, was mainly infusion related, and consisted of transient fever, chills, nausea and vomiting, and hypotension. Tumour necrosis factor alpha and IL-6 levels increased timely related to the side effects with a maximum after 2 and 4 h, respectively. Interestingly, toxicity appeared not to be dose related, in line with comparable peak levels of IL-6 and TNF-*α* in all cohorts above 0.35 mg m^−2^. Despite high peak levels of IL-6 and TNF-*α*, exceeding 300 pg ml^−1^, side effects were only moderate, which might be due to the absence of IL-1 and IFN-*γ* (Waage *et al*, 1989), and to antagonistic effects of an early release of anti-inflammatory cytokines such as IL-10. Furthermore, G-CSF might contribute to an enhanced anti-inflammatory cytokine response (Hartung *et al*, 1995). Concomitant administration of MDX-H210 and G-CSF seems to be safe in our study as well as in a multidose trial with MDX-H210 (Pullarkat *et al*, 1999). Interestingly, the side effects in our study were similar to other MDX-H210 studies, with other cytokines such as IFN-*γ* or GM-CSF (Posey *et al*, 1999; Lewis *et al*, 2001). On the other hand, the addition of G-CSF led to unacceptable toxicity in combination with an anti-Fc*γ*RI × anti-EGFR BsAb (MDX-447) (Pfister *et al*, 1999).

As MDX-H210 is designed to trigger phagocytic cells, the transient drop in ANC and monocytes was not surprising. Additional haematological changes were a more than 10-fold increase of circulating PMN, due to Filgrastim, and all PMN expressed Fc*γ*RI. Pulmonary toxicity, resulting from entrapment of activated Fc*γ*RI-positive PMN in the lungs, was considered to be a potential risk from G-CSF administration. Therefore, in some patients imaging with ^111^In-labelled granulocytes was performed. However, we did not observe increased clinical pulmonary toxicity nor did we observe abnormal accumulation of PMN in the lung. Three patients were retreated because of stable disease after 30 days. Strikingly less side effects were noted in these patients. This desensitisation phenomenon was also reported in other clinical trials with BsAb or mAb (Maloney *et al*, 1997; Dillman, 1999; Posey *et al*, 1999; Pullarkat *et al*, 1999).

In patients with advanced cancer, the cytotoxic capabilities of effector cells are often impaired (Brittenden *et al*, 1996). In contrast, granulocyte function was not diminished in our patients with regard to phagocytosis and oxidative burst, and was even further enhanced during G-CSF treatment. Isolated PMN were highly cytotoxic *in vitro* in the presence of MDX-H210, concomitant with the induction of Fc*γ*RI expression during G-CSF application. Maximum lysis was achieved at a concentration of 0.4 *μ*g ml^−1^, with reduced efficacy at higher doses, probably resulting from inhibition by monomeric binding of MDX-H210 to effector and tumour cells (Stockmeyer *et al*, 1997). Plasma concentrations of MDX-H210 exceeding 1 *μ*g kg^−1^ were already found in the 3.5 mg m^−2^ cohort; with increasing peak levels and AUC up to 200 mg m^−2^ with a serum half-life of 4–10 h, increasing to 17 h at doses of 200 mg m^−2^. Granulocytes and monocytes of patients treated at the 200 mg m^−2^ cohort documented complete saturation of Fc*γ*RI by BsAb for up to 4 days. These ‘armed’ effector cells are functionally active with high cytolytic activity in an ADCC assay without additional MDX-H210.

*In vivo*, erythema of involved skin areas in three patients and pain at tumour sites after antibody infusion suggest the induction of an inflammatory response within tumour lesions. In addition, biopsies from a metastatic skin lesion revealed infiltration with monocytes and PMN. Despite the fact that we were able to achieve adequate plasma concentrations for up to 4 days, it was unlikely that optimal concentrations of MDX-H210 were achieved within the tumour, since unfortunately we could not detect MDX-H210 in histological sections (data not shown), and failed to demonstrate tumour imaging using technetium-labelled MDX-H210. In contrast, good imaging of the Fc*γ*RI-positive effector cell pool was seen. One way of overcoming this limitation, probably caused by the preferential binding of MDX-H210 to effector cells, could be to either start Filgrastim after the administration of MDX-H210 to reduce the accessible Fc*γ*RI-binding sites, or by altering the pharmacokinetic properties of MDX-H210 to achieve high blood levels over a longer period of time. One way could be repetitive doses of MDX-H210, which might be limited by rapid HABA induction seen in more than half of the patients in this study. A BsAb construct with two fully humanised parts could help to overcome this problem. Compared to complete IgG antibodies, MDX-H210 has a very short half-life that cannot be fully explained by the reduced size of about 100 kDa. Since MDX-H210 lacks binding sites for the neonatal Fc receptor FcRn, which is critical for the serum half-life of IgG, engineered BsAb with altered affinities for FcRn might also help to increase the serum half-life (Ghetie and Ward, 2000). Another possibility of improving the off rate of the antibody from tumour sites is to design BsAb with an increased affinity for the tumour target, although a very high affinity could lead to impaired tumour penetration (Adams *et al*, 2001).

The intention of this trial with BsAb MDX-H210 was to utilise PMN as additional effector cells for breast cancer immunotherapy. This large cell population can be expanded and activated by G-CSF, which also induced Fc*γ*RI expression. Fortunately, concomitant treatment with MDX-H210 and Filgrastim did not lead to limiting toxicity. Although no objective response could be documented in these heavily pretreated patients with progressive breast cancer, biological effects were noted. Thus, MDX-H210 can be safely administered in combination with Filgrastim, and leads to highly efficient, expanded effector cell populations that may well have a significant therapeutic impact when employed in an optimised extended treatment schedule.
